# The role of macrophages in classic Marek's disease.

**DOI:** 10.1038/bjc.1973.85

**Published:** 1973-07

**Authors:** J. C. Campbell


**THE ROLE OF MACROPHAGES IN CLASSIC MAREK'S DISEASE.** J. C. Campbell. Cancer Research Campaign Unit, Department of Veterinary Pathology, Bush House, Milton Bridge, Midlothian.

Marek's disease is a contagious lymphoproliferative condition of chickens caused by a herpesvirus Type B and exhibits neural and visceral forms. Cells involved are mainly lymphocytes and plasmacytes. Lesions may terminate as lymphomata.

Indirect and direct immunofluorescence of cryostat sections and serosal spreads show antigen mainly confined to histiocytes, though vascular endothelium and neurilemma are frequently positive and lymphoid foci occasionally so. Fresh buffy coat preparations show positively staining monocytes which metamorphose in culture to motile macrophages. Lymphocytes adhere to these, forming conspicuous immunofluorescent clusters. Intercellular bridging between macrophages leads to the formation of immunofluorescent-positive polykaryocytes. Lymphocytes containing macrophage-endowed viral genome respond to the blastogenic effect of phytohaemagglutinin *in vitro* and naked nucleoplasmic virions appear

(Campbell and Woode, *J. med. Microbiol.*, 1970, **3**, 463). Intracranial injection of lymphocyte cultures containing transformed cells has produced cerebral lymphomata in chickens.


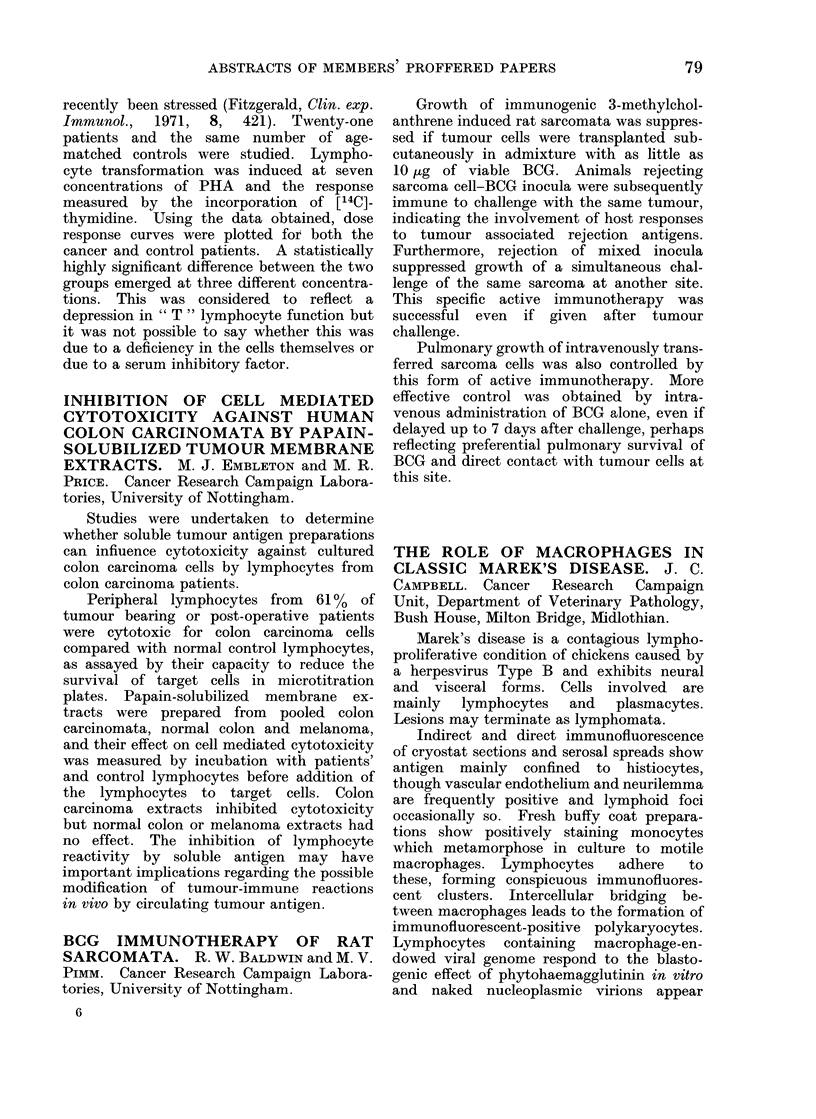

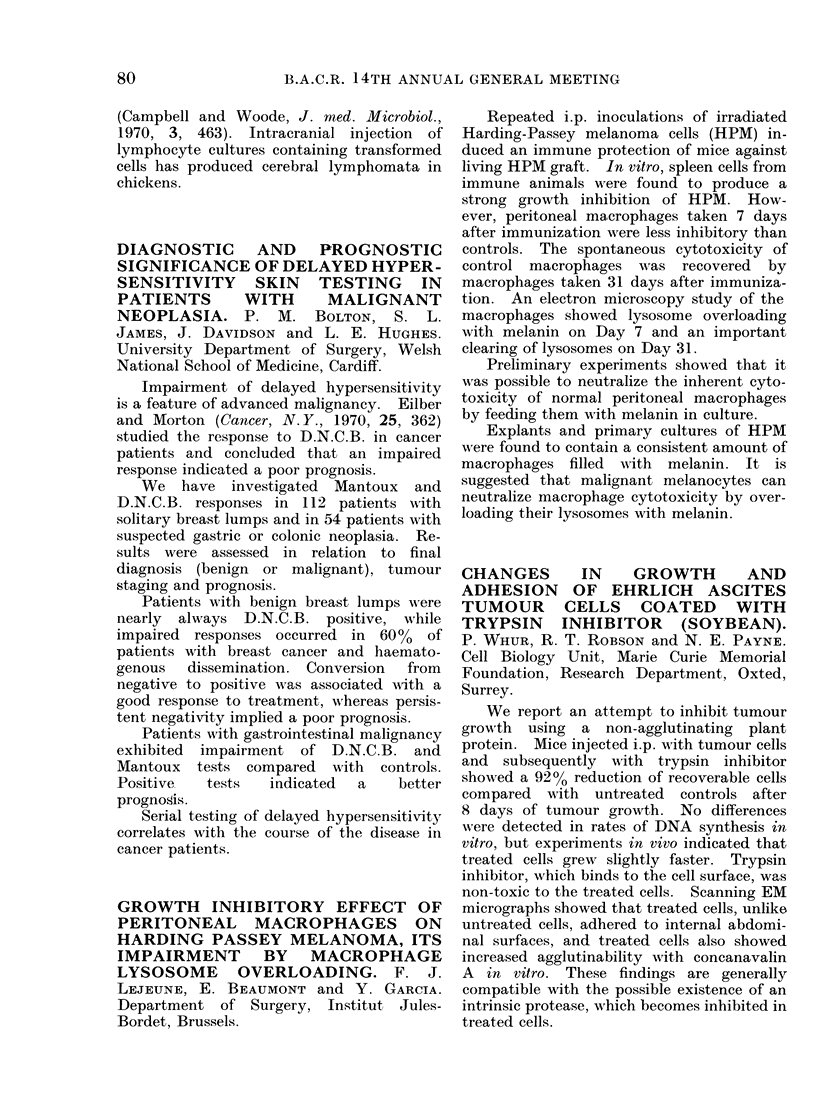


## References

[OCR_00037] Campbell J. G., Woode G. N. (1970). Demonstration of a herpes-type virus in short-term cultured blood lymphocytes associated with Marek's disease.. J Med Microbiol.

